# Extended Reality for the Clinical, Affective, and Social Neurosciences

**DOI:** 10.3390/brainsci10120922

**Published:** 2020-11-30

**Authors:** Thomas D. Parsons, Andrea Gaggioli, Giuseppe Riva

**Affiliations:** 1 iCenter for Affective Neurotechnologies (iCAN), Denton, TX 76203, USA; 2 Computational Neuropsychology and Simulation (CNS) Laboratory, University of North Texas, Denton, TX 76203, USA; 3 College of Information, University of North Texas, Denton, TX 76203, USA; 4 Humane Technology Lab, Università Cattolica del Sacro Cuore, 20123 Milan, Italy; andrea.gaggioli@unicatt.it (A.G.); giuseppe.riva@unicatt.it (G.R.); 5 Applied Technology for Neuro-Psychology Laboratory, Istituto Auxologico Italiano, 20145 Milan, Italy

**Keywords:** social neuroscience, clinical neuroscience, affective neuroscience, presence, virtual reality, extended reality, neuropsychology

## Abstract

Brain science research often involves the use of low-dimensional tools and stimuli that lack several of the potentially valuable features of everyday activities and interactions. Although this research has provided important information about cognitive, affective, and social processes for both clinical and nonclinical populations, there is growing interest in high-dimensional simulations that extend reality. These high-dimensional simulations involve dynamic stimuli presented serially or concurrently to permit the assessment and training of perceivers’ integrative processes over time. Moreover, high-dimensional simulation platforms can contextually restrain interpretations of cues about a target’s internal states. Extended reality environments extend assessment and training platforms that balance experimental control with emotionally engaging background narratives aimed at extending the affective experience and social interactions. Herein, we highlight the promise of extended reality platforms for greater ecological validity in the clinical, affective, and social neurosciences.

## 1. Introduction

Brain scientists have historically created and validated parsimonious low-dimensional stimulus presentations using basic technologies (i.e., static stimuli; limited interactivity; text-based vignettes). It is important to note that essential findings have resulted from these low dimensional stimulus presentations. Moreover, there are times when these stimulus presentations capture the information needed to advance brain science. That said, the parsimony offered by low-dimensional stimulus presentations may not always reflect much higher-dimensional social, affective, and cognitive constructs. There may be times that low dimensional stimulus presentations offer diminished interpretations of complex phenomena.

**Figure 1:**
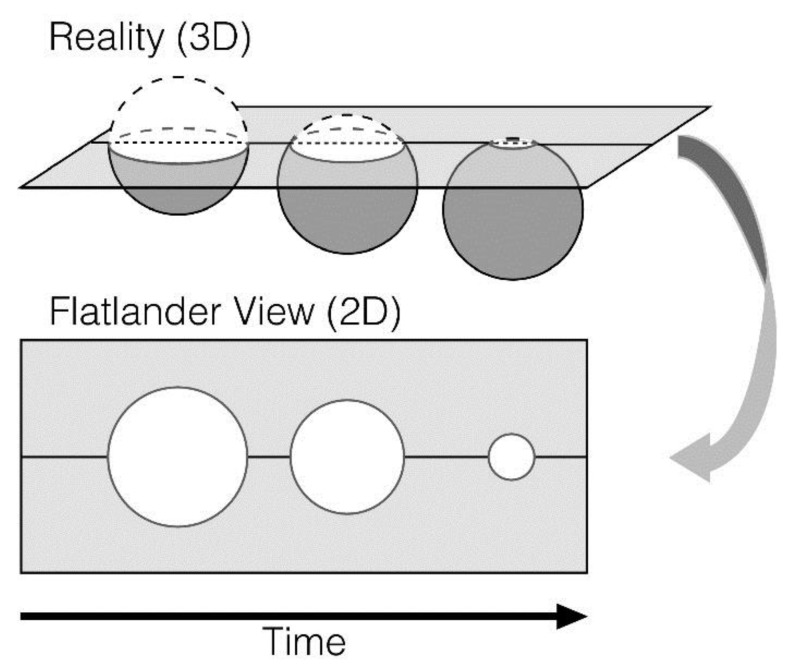
A. Square cannot perceive his world as anything other than two dimensional. (Reprinted by permission of the publisher).

Low-dimensional stimulus presentations in brain science reflect the Flatland perspective in Edwin Abbot’s [1] text on dimensionality and perception. In the work, A. Square (Abbot’s narrator) is a Flatlander who can only perceive two dimensions. A conversation with a “Stranger” (a sphere) leads A. Square to perceive the actual complexity and high dimensionality of the world. The tragedy of this story is that A. Square’s communication of newly held understandings are deemed heretical and lead to incarceration. For brain scientists, low dimensional stimulus presentations may result in simplified explanations of complex phenomena, which may in turn limit the development, validation, interpretation, and communication of useful models of human cognition, affect, and social interactions. According to Jolly and Chang [2] this “Flatland fallacy” can be overcome by formalizing psychological theories as computational models with the capacity for producing detailed predictions about neurocognition and/or behaviors (see Figure 1).

For Jolly and Chang, the Flatlander’s restricted viewpoint (bottom of the figure) leads to perception of a three-dimensional object (sphere) as varying sizes (increasing and decreasing radii) of a circle. This object (top of the figure) is merely progressing across a lower-dimensional plane. The Flatland (low-dimensional) perspective limits the Flatlander’s perception of reality. Comparably, brain scientists may at times imprecisely determine that a low level of dimensionality comprehensively explains cognitive, affective, and social phenomena.

There is increasingly discussion of the need for models established from high-dimensional stimulus presentations that better reflect the reciprocal relations among people and the environments in which they carry out everyday activities [3,4]. In the clinical, social, and behavioral sciences, there is interest in enhanced understanding of complex and dynamic interactions involved in the assessment of the brain’s processes in environmental and social systems [5,6]. Moreover, for the social, cognitive, and affective neurosciences there is growing attention to the development of high-dimensional tools for assessing and modeling brain functions using high-dimensional presentations of environmentally relevant stimuli [6,7].

Our cognitive, affective, and behavioral processes often involve social interactions and the negotiation of multifaceted environments. Brain scientists have both observed and explained social, cognitive, and affective processes throughout the history of the discipline. Brain scientists aim to enhance understanding of the consistent interconnections of specialized brain networks involved in social, cognitive, affective processes and behaviors [8]. Technological updates are needed for questions that can be best answered with high-dimensional stimulus presentations. Brain scientists have long used high dimensional technologies like functional neuroimaging for real-time observations of brain functioning. An additional high-dimensional tool is virtual reality that offers enhanced stimulus presentations and interactivity. Observing people as they interact in adaptive virtual environments holds promise for redefining previous understandings of social, cognitive, affective, and behavioral functions [9]. While the use of virtual environments in the brain sciences (e.g., social, cognitive, affective, clinical neurosciences) has not found the same level of adoption as psychophysiology and neuroimaging, virtual reality (VR) platforms can offer some advantages. For example, VR allows for the development and application of simulations of everyday activities and interactions. In this paper, we aim to discuss the ways in which high-dimensional VR stimuli and interactive platforms can amplify our understanding of significant characteristics found in real-world activities. An additional advantage is the naturalistic stimuli found in VR platforms. In field-based or natural experiments, it is difficult to control various extraneous variables that are problematic (even impossible) to control. A VR platform, however, is able to simulate naturalistic environments and maintain control of the environmental stimuli. Moreover, virtual environments may offer platforms that balance the need for high-dimensional simulations of real-life activities and interactions (i.e., ecological validity) with experimental control [10,11].

While brain science research can deliver stimuli via low-dimensional tools (paper-and-pencil assessments; text-based or audio-based vignettes; non-interactive videos), there is an increasing interest in using high-dimensional virtual reality (VR), augmented reality (AR), and mixed reality (MR) platforms (include both AR and VR). For VR platforms a headset is used that obscures visual stimuli from the real-world. In AR platforms, users are able to view the real-world with overlays of virtual elements. Platforms using MR involve a combination of VR and AR with both virtual and real-world features. Extended reality (XR) is an umbrella term that includes these three high-dimensional simulation types. These XR platforms can also dramatically reduce some of the risks inherent in real-world situations by placing users in simulations instead of dangerous real-world situations.

Extended reality environments (e.g., virtual reality) offer methodologies for presenting high dimensional simulations of everyday activities. Herein, we discuss the promise of extended reality platforms for presenting high-dimensional and dynamic stimuli that can be used for assessment and training of social, cognitive, and affective aspects of real-world activities in both healthy and clinical populations. We discuss several studies that have used extended reality platforms to explore social, cognitive, and affective processes. While findings from brain science studies are emphasized throughout, a number of examples are proffered that reference behavioral performance assessment. While the review includes information on AR and MR platforms, the majority of XR research in brain science has involved VR. As a result, the review has an emphasis on VR throughout. Although this is not an exhaustive review, it does highlight research related to participants responding to high-dimensional social, cognitive, and affective stimuli in simulations that approximate real-world activities and interactions.

## 2. Clinical Extended Reality (XR) for Assessment and Training in Clinical Neuroscience

### 2.1. Assessments: Simulation-Based Neuropsychological Assessments

Assessment in clinical neurosciences (e.g., clinical neuropsychology) often use static low dimensional stimuli that represent technologies from the past century. These traditional neuropsychological assessments were often developed for non-clinical populations and later normed for use in clinical settings. Moreover, these low dimensional stimulus presentations are often ones that focus on abstract cognitive constructs. Neuropsychologists give these measures and then try to use them for predicting future performance on everyday cognitive functions. Burgess and colleagues’ [12] contend that this process is going in the wrong direction. Instead, development of neuropsychological assessments should involve: (1) the establishment of tasks that reflect real-world “functions” and (2) progress backward from directly observable everyday functions (e.g., behaviors) to the how action sequences lead to a given cognitive function. This “function-led” approach requires clinical neuroscience tools be developed and validated. For example, VR platforms offer high dimensional and function-led XR measures for clinical neuropsychological assessment batteries.

Part of the attraction of XR platforms is their potential for improving the ecological validity of neuropsychological assessments [13,14]. Given that XR platforms like VR offer accurate and controlled presentation of dynamic/three-dimensional perceptual stimuli, they can balance ecologically validity and experimental control. Moreover, the enhanced computation power increases the accuracy of neurobehavioral responses recording. Simulation technologies like VR offer distinctively ecologically valid tasks [15]. High dimensional XR platforms with immersive simulations offer improved stimulus presentations that replicate the distractions, stressors, and/or demands found in everyday activities. It is important to note that there are times when traditional neuropsychological assessments adequately reflect cognitive function. Further, low-dimensional stimulus presentations that are well validated offer advantage over less well validated virtual environments. That said, there are increasing efforts aimed at psychometric validation of virtual reality based neuropsychological assessments see [16,17,18,19] for quantitative meta-analyses. Once adequately normed, these virtual environments should offer additional understanding of the patient’s performance of activities of daily living.

### 2.2. Treatment: Presence-Inducing Embodied Experiences

In addition to assessment, extended reality platforms are increasingly applied to rehabilitation and training. The application of clinical XR to rehabilitation involves integrating XR technologies into successful treatments that involve a suitable blend and progression of tasks to recover functional capacities. Rehabilitation programs start in the clinic setting and may then proceed to home settings. Advances in XR technologies offer new possibilities for rehabilitation. The various options include VR, AR, MR, gamification, and telerehabilitation. Likewise, XR platforms can be used for training. Kaplan and colleagues [20] performed a quantitative meta-analysis to explore empirical results from studies of training transfer from VR, AR, and MR. Moreover, they aimed to ascertain whether XR-based training has the same efficacy as traditional training methods. Results revealed that throughout several studies in a number of fields, XR platforms were as effective for training as traditional methods. This work can be extended to XR platforms that offer an immersive, presence-inducing experience with a significant potential for mental health assessment and treatment [21]. For example, post-traumatic stress disorder [22,23,24] and specific phobias (e.g., cockroach phobia; arachnophobia) have been assessed and treated with AR, VR, and MR (see [25,26,27,28,29,30] for systematic reviews).

Clinical XR can be defined as high-dimensional human-computer interfaces that permit clinical neuroscientists to: (1) immerse patients in simulations of real-world tasks and (2) allows the patient to manipulate objects and interact with others. According to Slater [31,32], presence in virtual reality is “the extent to which people respond realistically within a virtual environment, where response is taken at every level from low-level physiological to high-level emotional and behavioural responses” (p. 3555). This vision, also known with the acronym RAIR—response-as-if-real—is the result of two different illusions (Slater [32]): place illusion (PI), the qualia of having a sensation of being in a real place, and plausibility illusion (Psi), the illusion that the scenario being depicted is actually occurring. As explained by Slater: “If you are there (PI) and what appears to be happening is really happening (Psi), then this is happening to you! Hence you are likely to respond as if it were real” (p. 3555). But why is this happening?

A possible explanation is that the mechanisms used in adaptive virtual environments for real-time adaptations and multisensory feedback and integration, are similar to the ones used by our brain to generate an internal models (or matricies) of our physical bodies and their surrounding environments [33], clinical XR reflects work conducted in the emerging paradigm of “predictive coding” [34,35], in which our bodily experiences are outcomes of inferential processes that involve brain-based predictions (priors of the predictive brain) about bodily states. In fact, to allow an effective interaction with the physical world, our brains create an embodied simulation of our body that reflects its expected future states (intentions and emotions). This simulation has three specific features [33]: (1) it is a simulation of sensory-motor experiences; (2) embodied simulations are based on the expectations of the subject (including social schemas, heuristics, and biases) and reactivated multimodal neural networks that produced (in the past) simulations of expected effects; and (3) embodied simulations regulate relative to (dis)agreements [36] among perceived sensory activities (perceptions) and the contents of the simulations used to predict the effects of the being in the world of the individual.

In summary, clinical XR technologies attempt to predict the sensory outcomes of the patient’s actions by delivering the same outcome expected by our brains in the real-world. As explained by Riva and colleagues [33]: “To achieve it, like the brain, the VR system maintains a model (simulation) of the body and the space around it. This prediction is then used to provide the expected sensory input using the VR hardware. Obviously, to be realistic, the VR model tries to mimic the brain model as much as possible: the more the VR model is similar to the brain model, the more the individual feels present in the VR world” (p. 89). These features allow clinical XR platforms (e.g., VR) to structure, augment, and/or replace the patient’s bodily experience in novel ways that can be used in clinical applications [37,38,39,40]. Further, clinical XR provides novel embodied paths to assess patient brain function [41] by directly targeting the processes underpinning real-world behaviors [42,43,44]. While this review cannot proffer an exhaustive set of clinical cases and cohorts, examples are highlighted to discuss clinical XR in terms of structuring multisensory bodily contents, augmenting multisensory bodily contents, and replacing multisensory bodily contents.

#### 2.2.1. Clinical XR: Structuring Multisensory Bodily Contents

Variations in the extent of one’s sensitivity and attentiveness to bodily signals and sensations are apparent across individuals [45]. Application of XR can improve levels of body awareness when it includes the integration of simulations with biosensor technologies and the same can be found in the integration of virtual environments with biofeedback. Such platforms can be utilized for assessment, control, and training of cardiovascular responses (e.g., heart rate), skin conductance (e.g., galvanic skin response), muscle tension (e.g., electromyography), and brain waves (e.g., electroencephalography [46,47]). The use of the sensor metrics is important because they offer metrics that normally not consciously available to the patient. Observation of these signals educates the patient on the need to adjust these measured signals in more desirable directions relative to the feedback provided by XR platforms (e.g., flow of waterfall adjusts according to the patient’s heart rate).

This approach can also be used to improve social communication, social interactions, and recognition of affective arousal. An example clinical application is in the treatment of autism [48]. The efficacy of clinical XR for people with autism has been explored in systematic reviews of AR [49] and VR [47]. In a recent systematic review of the usage of AR technology (e.g., smartglasses, mobile devices, video projection systems) with people with autism, revealed positive main results. Moreover, they found that social skills, communication, and cognitive skills improved with the AR usage [50]. The benefits of using clinical XR are apparent in studies showing that practicing difficult (or individually challenging) social interactions leads to less-anxiety [51,52]. Through the simplification and structuration of everyday social skills, patients can practice social interactions (i.e., finding another to sit with in a classroom) without the anxiety levels and/or fears of rejection that commonly accompany real-world social interactions. A similar approach has also been used to improve social cognition, social skills, and reduce social anxiety in schizophrenia [53] patients and obsessive-compulsive disorder [54,55].

#### 2.2.2. Clinical XR: Augmenting Multisensory Bodily Contents

The integration of biosensors, stimulation, and haptic devices into clinical XR offers the clinical neuroscientist with tools for mapping the contents of a given sensory channel to others (e.g., visual processing mapped to kinesthetic or to auditory processes), increase sensitivity, and/or replace the impaired channels [56]. For example, Ward and Meijer [57] developed a virtual sound experience that converted sensory information normally supplied to the visual field into an auditory representation. In a different study, Suzuki and colleagues [58] combined feedback of interoceptive information (heart rate) with computer-generated augmented reality to produce a “cardiac rubber hand illusion”. Their results suggest that the feeling of ownership of the virtual hand can be heightened by cardio-visual feedback in time with the actual heart rate. This supports to utilization of this approach for improving affective regulation.

Another example of this approach is the “avatar therapy”, originally developed for patients with psychosis [59], and also suggested for treating depression [60]. In this therapy the patient creates a virtual avatar that extends his/her boundaries. Specifically, the patient externalizes his/her auditory verbal hallucinations to this avatar through seeing and hearing it performing speeches that the patient would otherwise have attributed to the verbal hallucinations. In this way avatars allow to externalize the self-criticism and other negative automatic thoughts frequently reported by patients with psychosis and depression.

A further example of this approach is the combination of XR and biofeedback to foster empathic abilities in both clinical (i.e., psychosis) and non-clinical subjects [61]. By merging the experience of the avatar of another individuals and the information about his/her internal states it is possible to step into the shoes of the other individual [62]. A recent study, used interpersonally shared biofeedback (visualised respiration rate and frontal asymmetry of electroencephalography, EEG) to enhance synchrony between the users’ physiological activity and perceived empathy towards the other during a compassion meditation exercise carried out in a social VR setting [63].

#### 2.2.3. Clinical XR: Replacing Multisensory Bodily Contents

Clinical XR allows for different types of synthetic bodily experiences including the patient’s body is substituted by a virtual body [64]. Similar to the movie “Being John Malkovich”, patients in clinical XR platforms that allow body swapping can experience another individual’s perspective, as well as see, hear, and touch what the other experiences. An example of this application from a social XR perspective is “The Machine To Be Another” (TMTBA). The TMTBA uses real time video to allow two people to exchange bodies in real time [65]. In addition to perspective swapping (via visuomotor and visuotactile synchronicity) the TMTBA can be utilized for presenting real-life narratives from various individuals acting as performers (experiencing their perspective).

Clinicians can leverage such research in their work with patients to develop clinical XR platforms and apply them in assessment and treatment. For example, this approach has been used to correct a faulty body representation and or affective response in patients with eating and weight disorders. Eating disorders may reflect deficits in multisensory bodily representations (processing and integration of signals) [66,67]. Specifically, the multisensory body integration deficit may impair a patient’s capacities (1) for identifying the pertinent interoceptive signals for predicting potentially agreeable (or aversive) outcomes and (2) for modifying/correcting the autobiographical allocentric (observer view) memories of body-related events (self-objectified memories).

Clinical XR platforms allow clinicians to target impaired multisensory body integration through two different strategies: (1) reference frame shifting [68,69] and (2) body swapping [70,71]. These strategies can be integrated with classic cognitive behavioral training (CBT) paradigms. The first method, “reference frame shifting”, has been successfully used in clinical trials [72,73] to modify the individual’s bodily self-consciousness through focusing and reorganizing body-related memories [74]. To achieve this goal, the patient re-experiences in VR a negative situation related to the body (e.g., teasing) both from first-person (egocentric) and third-person perspectives (e.g., seeing and supporting his/her avatar in the VR world). The clinician typically asks the patient to report in detail their virtual experience and the feelings associated with it. Moreover, the therapist educates the patient on ways to manage these feelings using various cognitive practices. In body swapping [75], virtual environments can be used for inducing illusory feelings of ownership of a virtual body with a dissimilar shape and/or size to rectify the dysfunctional body representation and lessen body-related anxiety and dissatisfaction [38,76,77,78,79,80,81].

## 3. Affective Extended Reality (XR), Affective Computing, and Affective Neuroscience

Emotional processes are important for perception, learning, judgements, decision-making, social interactions, and several additional functions for human functioning. Affective states (e.g., anger, sadness, fear, joy) alter brain activities [82,83] and are connected to neurophysiological interactions between primarily cortical-based cognitive processes and subcortical valence and arousal systems [84]. Neuroimaging data point to the significance of affect-related brain regions (e.g., orbitofrontal cortex, the dorsolateral prefrontal cortex, cingulate, hippocampus, insula, temporal regions, and amygdalae) and networks in forming emotions [85,86,87].

High-dimensional XR platforms (e.g., VR) may enhance research in affective neuroscience. For example, VR has been used for assessment and training of various aspects of affective arousal and emotional regulation [27,28,29]. A bonus of XR platforms is that they can be used in research that could not safely be performed in when real-world settings that are too hazardous, costly, and/or time consuming. Interactive virtual environments have been found to be especially useful for presenting arousing stimuli to elicit psychophysiological and behavioral responses [88,89]. The VR platforms provoke a sense of “presence” (feeling of being there) in participants and has potential for evoking affective arousal in affective neuroscience environments. Researchers have found that the affective arousal stemming from immersive VR can significantly impact a participant’s sense of presence [90], feelings of anxiety [91], valence [92], self-compassion [93], arousal in simulated natural environments [94], and various moods in social environments with virtual human avatars [95].

An area of confluence for affective neuroscience and high dimensional tools is affective computing, which uses implicit measures to examine human responses [96]. Affective computing uses automatic quantification and affect recognition using implicit measures. In particular, it can be applied to affective neuroscience research when investigating relations between emotions and decision-making. Affective computing makes use of biometric signals (neuroimaging; psychophysiology; speech emotion estimation; facial expressions) and machine-learning algorithms for automated classification of affect. Many signals have been used, such as voice, face, neuroimaging and physiological [97].

The two primary approaches to affect elicitation in affective neuroscience (as well as affective computing) are active and passive. The active approach involves direct influence of participants, including dyadic interactions [98], social interactions [99], and behavioral manipulation [100]. The passive approach typically presents low-dimensional external stimuli (standardized on the basis of valence and arousal [101]) via images (e.g., International Affective Picture System), sounds (International Affective Digitalized Sound System), and/or video clips [102]. While a good deal of knowledge has come from these approaches, they are limited in their low dimensional stimulus presentations, lack of interactivity, and they are non-immersive. This may result in very limited levels of presence in the participants. As a result, these low dimensional stimulus presentations do not evoke in the participants a feeling of “being there”. This lack of presence limits affective arousal. Moreover, these approaches are non-interactive, which limits affective arousal. Virtual environments offer simulations of real-life scenarios that are immersive and interactive [103]. Affective computing paradigms that include VR can be used for affective arousal. When combined with machine learning, implicit virtual reality-based measures can lead to the development of automatic affect recognition models [104].

### Affective Interactions Using VR: The Link between Presence and Emotions

One of the main features of XR is that virtual events may arouse the same responses and feelings as real-life events. In their classic study, Slater and colleagues [105] simulated Stanley Milgram’s 1960s experimental approach using virtual reality. Participants were instructed to administer a sequence of word association memory trials to an unknown (i.e., stranger) female virtual human. Participants were instructed to administer an “electric shock” to the virtual human when she gave an incorrect response. Moreover, the participants were instructed to increase the voltage each time an incorrect response was given (by the virtual human). With each administration of an “electric shock”, the virtual human responded with communications of increasing discomfort and protests. Eventually, the virtual human demanded termination of the experiment. Results from their study revealed that despite the reality that all participants knew for certain that neither the virtual human nor the electric shocks were real, the participants still inclined to react to the virtual human’s apparent suffering as if it were happening in real-life. As noted in the text [105]: “In the debriefing interviews many said that they were surprised by their own responses, and all said that it had produced negative feelings—for some this was a direct feeling, in others it was mediated through a “what if it were real?” feeling. Others said that they continually had to reassure themselves that nothing was really happening, and it was only on that basis that they could continue giving the shocks”.

The relationship of affective arousal and presence have been manipulated in various studies. For example, in a study by Bouchard and colleagues [106], adult participants with snake phobias were immersed into a virtual environment and experimenter induced anxiety by manipulating participant apprehension. A single-item measure of presence was used. Findings revealed significantly higher levels of reported presence in participants reporting feeling anxious during the immersion. In another study, Baños and colleagues [107] measured presence for both virtual and imaginary scenarios. Participants were randomly assigned to either imagined or virtual environment conditions. Each participant’s subjective sense of presence was assessed at three time points (beginning, middle, and end). Findings reveal that the participants in “imagery” spaces condition reported a decreased sense of presence. This is in contrast to the increased sense of presence found in participants experiencing the “virtual” spaces condition. In a further study, Michaud and colleagues [108] experimentally manipulated the sense of presence in a sample of people with acrophobia taking an elevator ride and performing tasks on a scaffold outside of a 15-story building. Immersion in the high-presence virtual environment resulted in significantly higher anxiety when compared to a low-presence setting. On the one hand, the sense of presence was greater in the “affect inducing” environments. However, on the other hand, the affective state was impacted by presence level. Chirico and Gaggioli [109] have also investigated the extent to which emotions produced during VR immersion are related to those produced in real life. Findings suggest that affective reactions found in virtual and natural conditions were only significantly different for anger (significantly higher in the natural condition) and amusement (significantly higher in the virtual condition). No significant differences were found between virtual and real conditions on sense of physical presence or engagement dimensions of presence. That said, there were different correlation patterns between emotions and presence were after in vivo and in virtuo exposure. Additionally, Cadet and colleagues [110] investigated the relation of emotion to memory in virtual experiences, as well as the interaction with immersion and sense of presence. Findings suggest that affective experiences in virtual environments prompt greater levels of presence than neutral ones. Moreover, both immersion and affect can influence memory. The effect of affect on the sense of presence, however, has greater importance in lower immersion platforms. These findings comport well with findings from Schubring and colleagues [111] study that compared the effects of high-dimensional VR presentations with lower dimensional (two dimensional) stimulations. Findings suggest that overall VR revealed more robust effects for emotional and attentional processes when compared to conventional 2D stimulation.

These findings underscore the manifestation of bi-directional relations between presence and affective responding [112]. As summarized by Diemer and colleagues [113]: “On the one hand, fear-related elements in VR are input cues to the fear network—as proposed in emotional processing theory—and might thus directly enhance emotional arousal… On the other hand, however, emotionally relevant perceptual stimuli and information enhance a VR environment, making it more interesting, appealing to attention and ultimately, increasing, at least initially, arousal—irrespective of the emotional valence of the stimuli in question”. However, there is the other hand, which has higher levels of presence leading to larger levels of affective responding. As a result, researchers wanting to developing applications for manipulating affective arousal will want to focus on XR environments capable of induce a high feeling of presence (e.g., psychotherapy). There may be other situations, however, when the researcher is interested in increasing affective arousal for a heightened level of presence and reality judgment. In such situations, the focus would be on developing relevant XR platforms with intellectually and/or emotionally significant content. An example can be found in Gorini and colleagues’ [114] study that compared a sample of urban Mexican city-dwelling participants with a sample of rural village-dwelling participants. Findings revealed that exposure to a relaxing virtual environment had different physiological and psychological impacts on the participants relative to their cultural and technological backgrounds.

Further evidence can be found in Bouchard and colleagues’ [115] investigations of presence using a virtual environment designed for treating specific phobias. In both conditions, participants were immersed in the same VR platform containing a rodent. However, one condition deceived participants and led them to believe that they were actually immersed (in real time) in a physical room containing the rodent. This deception involved a blend of mixed videoconference and graphic displays, as well as misleading instructions declaring that participants were “currently living in the real room”. Significantly higher levels of presence were found in situations where participants were told they were seeing the “real” room (projected in the head-mounted display in real time) [93]. These results provide further evidence that manipulating presence can result in cognitive appraisals and influence both emotions and presence in VEs where the objective properties have not been altered [116].

## 4. Social Extended Reality and Social Neuroscience

Extended reality platforms can also be added to research undertaken by brain scientists who are interested in what has been called “Social Brain” research, which emphasizes the neurocognitive skills inherent in social activities and interactions [117]. In this research, ”Social Cognition” and social competencies are quantified and modeled. There is increasing interest in the potentially limited generalizability of social neuroscience studies using low dimensional stimuli (static; non-interactive; text-based vignettes). How well do these findings from low dimensional stimulus presentations generalize to the social cognitions found in everyday activities? To alleviate these potential limitations, Zaki and Ochsner [7] proffer three important ways that real-life social data differs from controlled laboratory stimuli. The first is multimodal inclusion of visual, semantic, and prosodic information. The second is dynamic (serially or concurrently) stimulus presentations to participants over time. The third is contextual embedding of stimulus presentations of stimuli and environmental information so that participants can the internal states of others. Multimodal stimulus presentations offered enhanced ecologically valid to social neuroscience measures that allow for social mentalizing and interactions to occur in situations involving the convergence of multiple auditory, visual, and perception channels. Moreover, there is a need for greater emphasis upon high dimensional, dynamic, and interactive platforms that allow for investigations of participants as they consider and interact with social stimuli. Hence, interactivity is preferred as it better reflects the ways in which social judgement and interactions occur in real-world settings. Unfortunately, many social neuroscience approaches presented static and controlled stimuli to participant who must believe, imagine, and act “as if”’ they could modify the course of a social interaction. As a result, some current social neuroscience approaches would benefit from adding real-time dynamic and adaptive XR platforms that include augmented reality [118,119], virtual environments [120,121], and virtual human agents with complex cognitive architectures [122].

### 4.1. Social XR

Social neuroscience researchers can make use of XR platforms that allow multi-user communication and interaction in synthetic environments. In a review by Kim and colleagues [123], AR applications were categorized into six common application types: military, industry, healthcare, games, tours, and media. Social XR platforms using AR have been employed for reducing cognitive workload in collaborative tasks [124,125], enhancing communication among collaborators [126], increase mutual understandings [127]. Several studies investigating the efficacy of various systems, usability, and design, have emerged in the literature (see [128,129] for reviews). Hilken and colleagues [118] used social augmented reality to explore the ways in which social XR (i.e., social AR) supports shared decision making. They showed that optimum social AR designs with dynamic (i.e., high-dimensional) point-of-view sharing was associated with image-enhanced communicative acts increased comfort with advice giving and likelihood of adopting recommender’s advice. They argue that these results reflect a sense of social empowerment that stimulates a recommenders’ desire for an object and positive behavioral intentions.

In a social XR study using VR, Jonas and colleagues [130] developed a social VR application taxonomy that can be used when making design choices. The taxonomy is informed by prototypical and commercial applications found in the literature. This reveals the potential of most of these platforms for use in creating avatars, interacting with others (via a variety of embedded communication channels), and building virtual spaces. More advanced social VR platforms also offer the potential for sharing photos and videos, game play, and the integration of other social media. social neuroscientists can use these applications for observing interaction paradigms that go beyond the standard voice-based chat. Moreover, these advanced platforms can include biometrics and neuro-feedback (e.g., respiration rates; brainwaves [131]). Social XR involves common theoretical frameworks and extensions of XR to the realm of social reality. This is the next logical step in the evolution of this medium.

### 4.2. Social XR with Virtual Humans

For social XR systems to be relevant to social neuroscience research, they need to have the capacity for multiple social actors to interact in an immersive virtual space. Co-located social actors can include perceptible digital representations via avatars that behave in a manner that reflects the behaviors of the user in real-time [132]. Another co-location of social actors can be found in computer-generated virtual agents that are controlled by algorithms. An additional category of synthetic social entities are “avatar–agent hybrids” that are somewhat automated and to some extent guided by a human controller [133,134].

Simulated representations of real users are an important aspect of social XR platforms. These avatars allow users to choose (or construct) an appearance that resembles the user’s own real-life characteristics. Moreover, these avatars can communicate essential information about its personality and characteristics [135]. Users can also utilize audio filters and prerecorded audio to simulate and/or modify the sound of their voice. This extends the capacity for personalization. Visual representations via avatars can differ substantially depending on the platform [130]. For some social XR platforms, the avatars can be represented as full bodies. For others, a partial body (i.e., head, arms, and torso) may be presented. A growing body of research is revealing that a user’s simulated body can significantly alter perceptions of identity and environment [136,137,138]. As mentioned above, investigations of these altered perceptions aim at enhancing understanding of virtual embodiment in various therapies [139], wellbeing applications [140], and investigations into the ways in which the brain maps the body schema onto a virtual object to create an illusion of ownership. For example, experimental paradigms such as the “body swap” [141] and the “rubber hand” illusions [142] have been developed. In social XR, avatars are typically controlled through the tracking and translation of the user’s body actions onto the avatar in real time [143]. Users can ambulate avatars in virtual spaces in a manner that mimics actual walking. Furthermore, social XR allows for the generation of social presence through the use of autonomous (or semi-autonomous) virtual agents that feature anthropomorphic appearance. These social XR platforms can track and register gestures, body motion, and user gaze. They can also produce verbal and non-verbal social signals that enhance the human user’s experience of social realism [144].

An important feature of social XR platforms is that they often integrate typical web-based social networking capabilities into their fully-immersive platforms. This offers the social neuroscientist with “meta-social media” capabilities. Dzardanova and colleagues. [145] contend that social XR may be extended to social networking in that it allows users to engage in synchronous, interpersonal interactions in web-based environments. Several communication possibilities are afforded by current social XR platforms: voice mediated, text based, physical expressions, and biometrics with-adaptive feedback. Hence, social XR offers may realize enhanced capabilities and may eventually become the most advanced social computing platform [146].

A further implementation of social XR can be found in interactions between human users and virtual humans (see [147] for a review of this literature). In Kim and colleagues’ [123] examination of papers published (2008–2017) in the International Symposium on Mixed and Augmented Reality (ISMAR), they found an expanding trend in evaluative studies. Embodied agents have been found to increase social presence through the successful integration of virtual content with the real-world [148,149,150].

### 4.3. Presence and Co-Presence (Key Concepts/Theories)

Presence researchers often define the sense of presence as the subjective experience of “being there” while interacting in XR platforms. Presence has largely been developed from VR research and can be differentiated from immersion, which involves the properties of a VR system [151]. The definition of presence is difficult, as there are significant differences in philosophical, psychological, and/or technological perspectives. The relevance of presence is both theoretical and practical. The level of presence may enhance the efficiency of a simulation and influence a user’s task performance. That said, there is no conclusive evidence related to a positive causal relationship [152]. To some extent, this is due to the paucity of studies that have investigated this question.

Although presence represents complex psychological phenomena related to a user’s experience of a simulated space as “real”, the extent to which a user perceives a simulated other to be “real” can be understood as “social presence” [153]. According to Short and colleagues [154], social presence describes the degree of salience between two communicators across different media forms. For social XR, the construct of social presence aids the social neuroscientist in understanding the ways in which users perceive the presence of other social entities (living or synthetic) in simulated environments. Biocca has defined social presence as the “sense of being with another” [155], p. 456 and social presence is influenced by the extent to which a user perceives “the access to the intelligence, intentions, and sensory impressions of another” [156], p. 22.

Social presence is believed to be a needed experiential feature for mediated environments and offers several positive communication outcomes. Riva and colleagues [157] have placed social presence into a bio–psycho–social that includes three subprocesses that while phylogenetically different, are mutually inclusive:

- Other’s presence: the capacity for recognizing another’s motor intentions. This allows for intentional recognition and imitation of another. The better the user is at recognizing (within the sensorial flow) another’s motor intention, the better able is the user to carry out intentions that enhance survival;
- Interactive presence: the capability for recognition of motor and proximal intentions. This permits the user to identify the intension of others toward the user. The better the user is competent in recognizing (within sensorial flow) the motor and/or proximal intentions of others (direct towards the user), the greater the chances of successfully carrying out social actions that enhance survival;
- Shared presence: the capacity for recognizing motor, proximal, and distal intentions. This allows the user to identify when another’s intentions correspond to the user’s intentions. The better the user is at recognizing (within sensorial flow) when another’s intentions are the same as the user’s, the better the user will be at successfully initiating and maintaining collective intentions that call for a form of cooperation beyond mere coordination. This involves mutual understanding of the intentions of the others and increases the user’s chances of survival.

If the user is able to recognize distal intentions (shared presence), then the user can also understand motor intentions (other’s presence). This model postulates that the maximal social presence can be accomplished when these three layers are successfully integrated. Moreover, these three social presence levels are connected by concurrent effects on the user’s ability to interact socially. According to the Riva and colleagues, the ways in which interactions are experienced changes relative to the level of social presence experienced by the user.

In a systematic review (152 manuscripts), Oh and colleagues [147] identified significant factors that influence social presence in mediated environments. These factors were classified in three broad categories: (1) immersive qualities, (2) contextual differences, and (3) individual psychological traits. By “immersive qualities”, they mean aspects of presence that are related to a medium’s technological capacity for generating realistic experiences. Examples include visual representations of the user’s communication partner; the interactivity levels provided by virtual agents; haptic feedback; depth cues; audio quality, and type of display. Contextual properties do not refer to objective immersive attributes. Instead, they are context-related and individual qualities that impact the user’s subjective perceptions of being together with another. Examples include: the avatar’s personality/traits; agency (whether controlled by a human or an algorithm); proxemics (distance between interactants); task types; social cues about the presence of others; and identity cues. Oh and colleagues also distinguish “individual psychological traits” that include the users’ demographic variables (e.g., gender, age, and psychological variables). Findings from the systematic review suggest that both immersion (e.g., depth cues, audio quality, haptic feedback, and interactivity) and context (e.g., proxemics, identity cues, and the personality/traits of the virtual human) can be significant predictors of social presence.

Social XR offers the social neuroscientist with platforms with capacities for eliciting a deep sense of social presence [158,159]. Social XR platforms with adequate levels of social presence offer important tools for the study of social cognition and related alterations in neural activity [160]. Moreover, social XR platforms can be used for assessing socio-cognitive processes in which representations of the social environment are generated [161]. Moreover, the use of XR in social neuroscience may circumvent the methodological limitations found in naturalistic observations. Calabrò and Naro [121] contend that examination of social cognition using social XR offers a venue for experimental control in the examination of neurobiological processes involved in social cognition (e.g., recordings of the brain’s intrinsic electrical activity, neuroimaging, and physiological measures). This is important as extrinsic stimuli may fail to adequately represent real-life social encounters. Furthermore, social XR offers the social neuroscientist a platform that can be used for increasing feedback to the user’s brain via concentrated and repetitive presentation of task-oriented tasks that can enhance learning.

## 5. Potential Limitations of Social XR Platforms

While much of this review has considered the positive implications of XR platforms, there are some potential risks and limitations that should be considered when implementing this technology. This is significant in that adverse side effects may limit the applicability of XR platforms for certain cohorts (e.g., clinical populations; older age participants). Of note, are two common side effects associated with simulations: cybersickness and aftereffects.

### 5.1. Simulator Sickness

The first concern is simulator sickness (also known as cybersickness) that can occur when users are immersed in simulations. Simulator sickness is a disagreeable side effect of simulations that can include a group of symptoms: motion sickness, fatigue, headache, eye strain, and/or nausea [162,163]. In virtual environments, these symptoms have been found to occur alone or together ([164,165]; see [166] for a systematic review and meta-analysis). Sensory mismatch and postural instability are often suggested as potential causes. For example, Bos and colleagues [167] have suggested a “vertical mismatch” framework for communicating and forecasting visually-induced motion sickness. Such sensory conflict approaches view simulator sickness as a symptom of mismatches between (or within) the visual, vestibular, and somatosensory inputs.

### 5.2. Depersonalization and Derealization

Another concern raised in the literature, is that the effects of simulations are similar to symptoms found in dissociative disorders (depersonalization and derealization). Aardema and colleagues [168] contend that the effects of immersion in simulations may influence the user’s agency and responsibility. It is important to note that there is not a great deal of evidence that XR platforms (VR or otherwise) can cause such effects in most users. That said, some have raised concerns about the potential negative impacts associated with problematic video gaming and virtual reality use [169,170].

## 6. Discussion

Extended reality platforms are both advanced simulation tools and technologies capable of augmenting and transforming our experience—acting on the sensory, cognitive, and emotional components that make it up. On the other hand, the transformative potential of extended reality is sustained and continuously fueled by new discoveries in the clinical, cognitive, social, and affective neurosciences, and by the availability of a simulation technology that generates ever more realistic simulations. Extended reality platforms provide powerful tools to enhance assessment and treatment of a wide range of mental and neurological conditions. This potential is not limited to the obvious advantages provided by the (controlled and safe) simulation of “conventional” low-dimensional stimuli and situations, but extends to the possibility of altering and manipulating these situations in unprecedented ways, i.e., inducing the illusory feeling of ownership of a virtual body with a different shape to reduce body-related anxiety. The possibilities offered by for the clinical, affective and social neurosciences could be further extended thanks to the progressive convergence between simulation technologies, neural interfaces, mobile/wearable devices, artificial intelligence and robotics. The emergence of this “confluence paradigm” will lead to totally new forms of interaction between human and machine, characterized by an increasingly “symbiotic” relationship between people and digital technologies [171]. These developments will lead to XR tools that enable an even deeper alteration of human experience: a perspective that questions us with respect to the ethical implications of this technology [172]. Actually, the very same tools that are used to uncover the complex interplay among brain, mind, and bodily phenomena, assess morally relevant decision-making behaviors, or develop new treatments for mental and neurological conditions, could be easily applied to cause psychological or physical harm to others. Thus, it is important that technological and methodological advances in XR neuroscience are accompanied by an increased awareness and recognition of the ethical risks that are potentially involved.
